# Changes in gut microbiota after gastric cancer surgery: a prospective longitudinal study

**DOI:** 10.3389/fonc.2024.1533816

**Published:** 2025-01-22

**Authors:** Yuhua He, Shilin Gao, Lili Jiang, Jie Yang

**Affiliations:** Colorectal Cancer Center, West China Hospital, Sichuan University/West China School of Nursing, Sichuan University, Chengdu, Sichuan, China

**Keywords:** gastric cancer, gut microbiota, high-throughput sequencing, surgery, bacteria

## Abstract

**Background:**

This study was designed to characterize gut microbiota changes of the patients with gastric cancer before and after the gastrectomy during their hospital staying periods.

**Methods:**

16S ribosomal RNA (rRNA) gene sequencing was used to evaluate differences in gut microbiota among patients with gastric cancer before and after the gastrectomy by comparing gut microbiota α diversity, β diversity, and structure composition at different taxonomic levels.

**Results:**

A total of 120 fecal specimens were collected from 60 patients. There was no significant difference in Chao1 index, Shannon index, and Simpson index before and after gastrectomy (all *P* > 0.05). At the phylum level, the gut microbiota in the gastrectomy group showed less abundance of *Bacteroidota*, *Synergistota*, and *Verrucomicrobiota* but with higher abundance of *Campylobacter*, *Actinobacteria*, and *Bacillota*. At the genus level, the gut microbiota in the gastrectomy group showed less abundance of flora *Bacteroides*, *Faecalibacterium*, *Blautia*, and *Lachnospiraceae nk4a136* group but with higher abundance of *Campylobacter*, *Porphyromona*, *Finegordia*, *Dialist*, *Anaerococcus*, and *Corynebacterium.*

**Conclusions:**

There was no significant change in the diversity of intestinal flora before and after surgery. However, significant changes in the structure of intestinal flora before and after surgery were occurred.

## 1 Introduction

Gastric cancer (GC) is a global health issue, being the fifth most prevalent cancer and the third leading cause of neoplasm death worldwide (1). China is experiencing a transition in its cancer profiles, with greater incidence of cancers (2). Gastrectomy as a curative resection for GC aims to obtain complete histopathological clearance and involves radical resection of the primary site, as well as resection of affected lymph nodes and adjacent organs if necessary (3).

The human gastrointestinal (GI) tract is a complex micro-ecosystem inhabited by as many as 10^14^ microorganisms, including bacteria, fungi, viruses, and protozoa (4). There are more than 400 species of bacteria in the intestinal microecology of the human body, of which the predominant flora mainly includes *Bacteroides* and *Bacillota*, which account for more than 70% of the total number of bacteria, and other bacteria mainly include *Proteobacteria*, *Clostridium*, *Actinomycetes*, *Warts Microbacteria*, and *Cyanobacteria* (5). Different intestinal flora jointly maintain the dynamic balance of the micro-ecosystem by restricting each other and participate in the body’s energy conversion, metabolism, digestion, immune regulation, and intestinal mucosal barrier defense functions (6–8).

With the technology of 16S rRNA gene sequence analysis, the changes of intestinal flora in patients undergoing gastrectomy during the perioperative period are gradually revealed. An increased gut microbial diversity and an altered microbial composition in conjunction with the metabolic improvements were found after Roux-en-Y gastric bypass (RYGB) (9). Furthermore, colonization of germ-free mice with fecal material from RYGB-operated mice caused weight loss and reduced adiposity, providing evidence that RYGB-associated gut microbiota can improve host metabolism (10). These clinical studies have shown major changes microbiota after gastrectomy, whereas most of the subjects were patients with obesity or mice undergoing RYGB surgery.

Different from the previous studies, patients with GC face multiple clinical exposure factors during the perioperative period, including antibiotics, diet, anxiety, and hospital pathogens, which will affect the intestinal flora (11). However, gut microbiota changes in patients with GC in perioperative period are little understood. In addition, intestinal microbiota may also be further affected in patients with GC due to reconstruction of digestive tract and reduction of tumor burden (12). Liang et al. first reported the impact of radical distal gastrectomy on the fecal microbiota of patients with GC and found that radical distal gastrectomy had a significant impact on the intestinal microbiota community composition, mainly manifested in the changes in the relative abundance of *Achmania*, *Escherichia/Shigella*, *Lactobacillus*, and *Microbacillus* (11). However, the sample size of this study was only six cases.

This study was designed to characterize fecal microbiota shifts in GC before and after surgery during their hospital staying periods.

## 2 Materials and methods

### 2.1 Study design

This is a before-and-after study in the same patient, which is designed to characterize fecal microbiota shifts in GC before and after surgery during their hospital staying periods, and has been reported in line with the Strengthening the reporting of cohort studies in surgery (STROCSS) criteria.

### 2.2 Ethical approval

Research ethics were proposed by the Biomedical Ethics Committee of West China Hospital of Sichuan University. After reviewing of the hospital ethics committee, trained study research assistants accomplished informed consent process.

### 2.3 Study participants

Study participants were recruited from January 2021 to December 2021 at West China Hospital of Sichuan University. The sample size was determined according to the species accumulation curve. When the sample size increases, the curve tends to be flat, indicating that the sample size meets the requirements. Finally, 60 adult patients were enrolled. Participants provided a written informed consent. The inclusion criteria for participants were as follows: diagnosed with GC by pathological biopsy under gastroscopy before operation; underwent elective gastrectomy; without other metabolic diseases (diabetes, obesity, gout, etc.) and infectious diseases (HIV infection, etc.); and no distant metastasis of liver, lung, bone, etc. Exclusion criteria included the following: a history of GI surgery; administration of probiotics, macrobiotic live preparations, antibiotics, metformin, proton pump inhibitors, berberine, or laxatives in the past 3 months; and preoperative with intestinal inflammation, perforation, obstruction, and severe systemic disease. All patients followed the concept of Enhanced Recovery after Surgery management during perioperative period. All patients fasted for 6 h and abstained from drinking for 2 h before surgery. Non-diabetic patients can take carbohydrate drinks ≤400 mL 2~3 h before surgery. Patients were not given preoperative intervention treatment such as probiotics, macrobiotic live bacteria preparations, antibiotics, proton pump inhibitors, and berberine, laxatives that affect the intestinal flora. Surgeries are performed by doctors in the same medical group.

### 2.4 Fecal sample collection

Patients collect their own fecal samples at two time points. The first stool sample is taken the day before surgery. The second sample was from the first natural bowel movement after surgery (13, 14). Patients placed the fecal samples directly into clean, dry, and non-absorbent covered container tubes (approximately 1.0 g per tube). The samples were placed in a freezer at −80°C within 30 min of excretion. Samples were transported to the Yakult Central Institute at −20°C for analysis for analysis of α diversity, β diversity, and gut microbiota structure composition at different taxonomic levels.

### 2.5 DNA extraction and amplification

Bacterial DNA was isolated from the fecal samples using a MagPure Soil/Stool DNA LQ Kit (Magen, Guangdong, China) following the manufacturer’s instructions. DNA concentration and integrity were measured by a NanoDrop 2000 spectrophotometer (Thermo Fisher Scientific, Waltham, MA, USA) and agarose gel electrophoresis, respectively. PCR amplification of the V3-V4 hypervariable regions of the bacterial 16S rRNA gene was carried out in a 25-μL reaction using universal primer pairs (343F: 5′-TACGGRAGGCAGCAG-3′; 798R: 5′-AGGGTATCTAATCCT-3′). The reverse primer contained a sample barcode and both primers were connected with an Illumina sequencing adapter.

### 2.6 Library construction and sequencing

The amplicon quality was visualized using gel electrophoresis. The PCR products were purified with Agincourt AM Pure XP beads (Beckman Coulter Co., USA) and quantified using a Qubit dsDNA assay kit. The concentrations were then adjusted for sequencing. Sequencing was performed on an Illumina NovaSeq6000 with two paired-end read cycles of 250 bases each. (Illumina Inc., San Diego, CA; OE Biotech Company, Shanghai, China).

### 2.7 Bioinformatics analysis

Paired-end reads were preprocessed using Trimmomatic software (15) to detect and cut off ambiguous bases (N). It also cut off low quality sequences with average quality score below 20 using sliding window trimming approach. After trimming, paired-end reads were assembled using FLASH software (16). Parameters of assembly were: 10 bp of minimal overlapping, 200 bp of maximum overlapping, and 20% of maximum mismatch rate. Sequences were further denoised as follows: reads with ambiguous, homologous sequences or below 200 bp were abandoned. Reads with 75% of bases above Q20 were retained using QIIME software (version 1.8.0) (17). Then, reads with chimera were detected and removed using VSEARCH (18). Clean reads were subjected to primer sequences removal and clustering to generate operational taxonomic units (OTUs) using VSEARCH software with 97% similarity cutoff (16). The representative read of each OTU was selected using QIIME package. All representative reads were annotated and blasted against Silva database (version 132) using Ribosomal Database Project (RDP) classifier (confidence threshold was 70%) (19).

The microbial diversity in fecal samples was estimated using the α diversity that includes Chao1 index (20), Shannon index (21), and Simpson index. The Bray–Curtis distance matrix performed by QIIME software was used for weighted UniFrac non-metric multidimensional scaling (NMDS). The 16S rRNA gene amplicon sequencing and analysis were conducted by OE Biotech Co., Ltd. (Shanghai, China).

### 2.8 Statistical analysis

The difference of α-diversity index in different groups was compared by Wilcoxon rank sum test. Principal coordinate analysis was used to identify overall gut microbial composition between preoperative patients and postoperative patients based on Bray–Curtis dissimilarity index. Wilcoxon rank sum was used to test the different species of intestinal flora before and after the operation, and the boxplot map of different species at the level of phylum and genus was drawn.

## 3 Results

**Figure 1:**
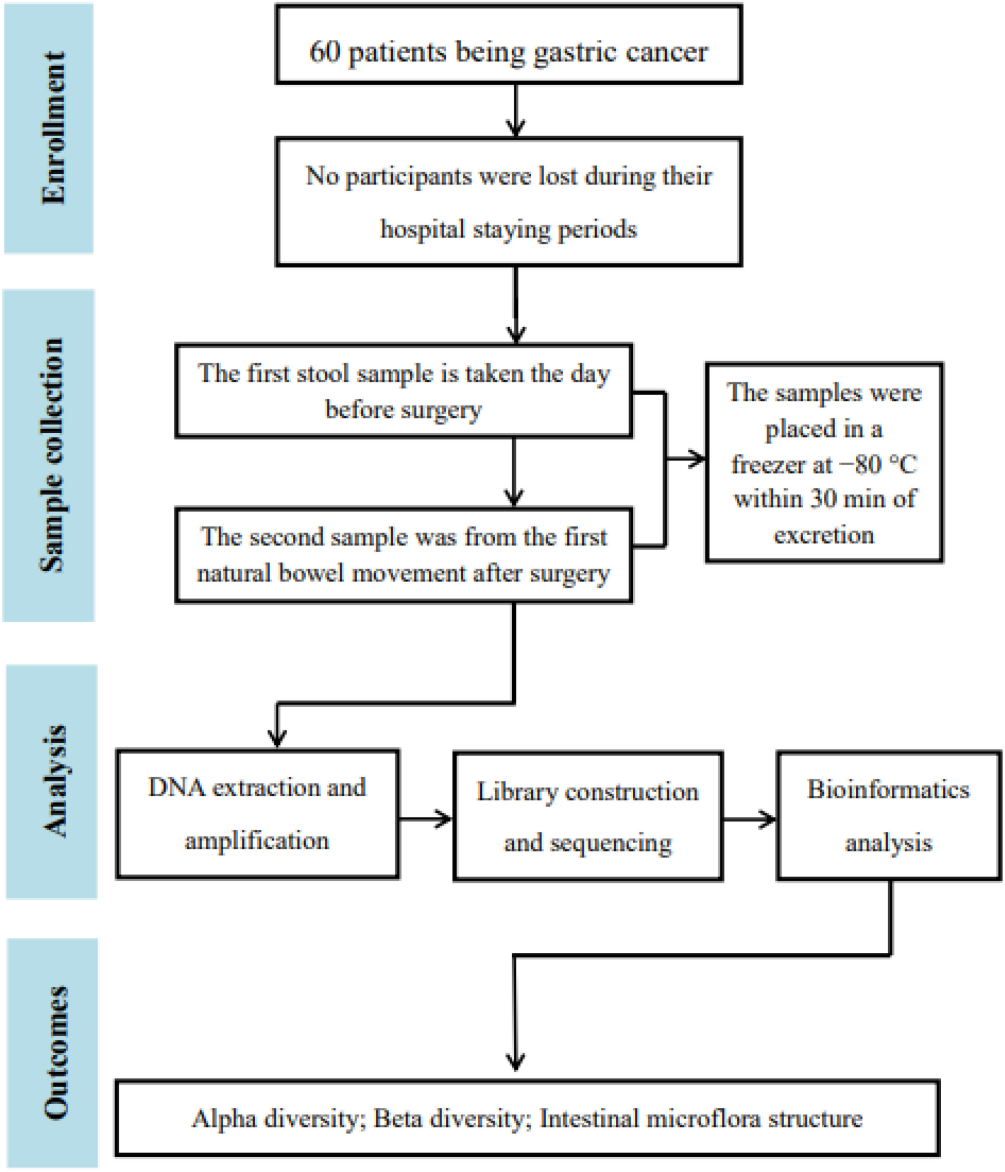
Flowchart of the study.

A flowchart of subject selection (a graphical representation of a process) was performed to illustrate the results of research and the screening process (**Figure 1**).

### 3.1 Collection of 16S results

A total of 120 stool samples from 60 patients were collected. Characteristics of these participants were seen in **Table 1**.

**Table 1:** The characteristics of participants.

Characteristics	Classification	N
Nation	The Han nationality	57
	Others	3
Sex	Male	36
	Female	24
Age, years, mean ± SD	65.10 ± 12.21	60
Preoperative comorbidity	No	5
	Yes	55
Preoperative neoadjuvant chemotherapy	Yes	28
	No	32
Gastric tube placed	No	60
Surgical approach	Laparoscopy	19
	Open	41
Intraoperative prophylactic antibiotics	Cefoxitin sodium, 1 g	32
	Cefmetazole sodium, 1 g	28
Intraoperative blood loss, mL, mean ± SD	52.75 ± 21.65	60
Digestive tract reconstruction	Billroth I	16
	Billroth II	21
	Roux-en-Y	23
Resection range	Proximal gastrectomy	16
	Distal subtotal gastrectomy	21
	Total gastrectomy	23
Surgical duration, hours, mean ± SD	4.72 ± 0.80	60
ASA	1	2
	2	28
	3	30

**Figure 2:**
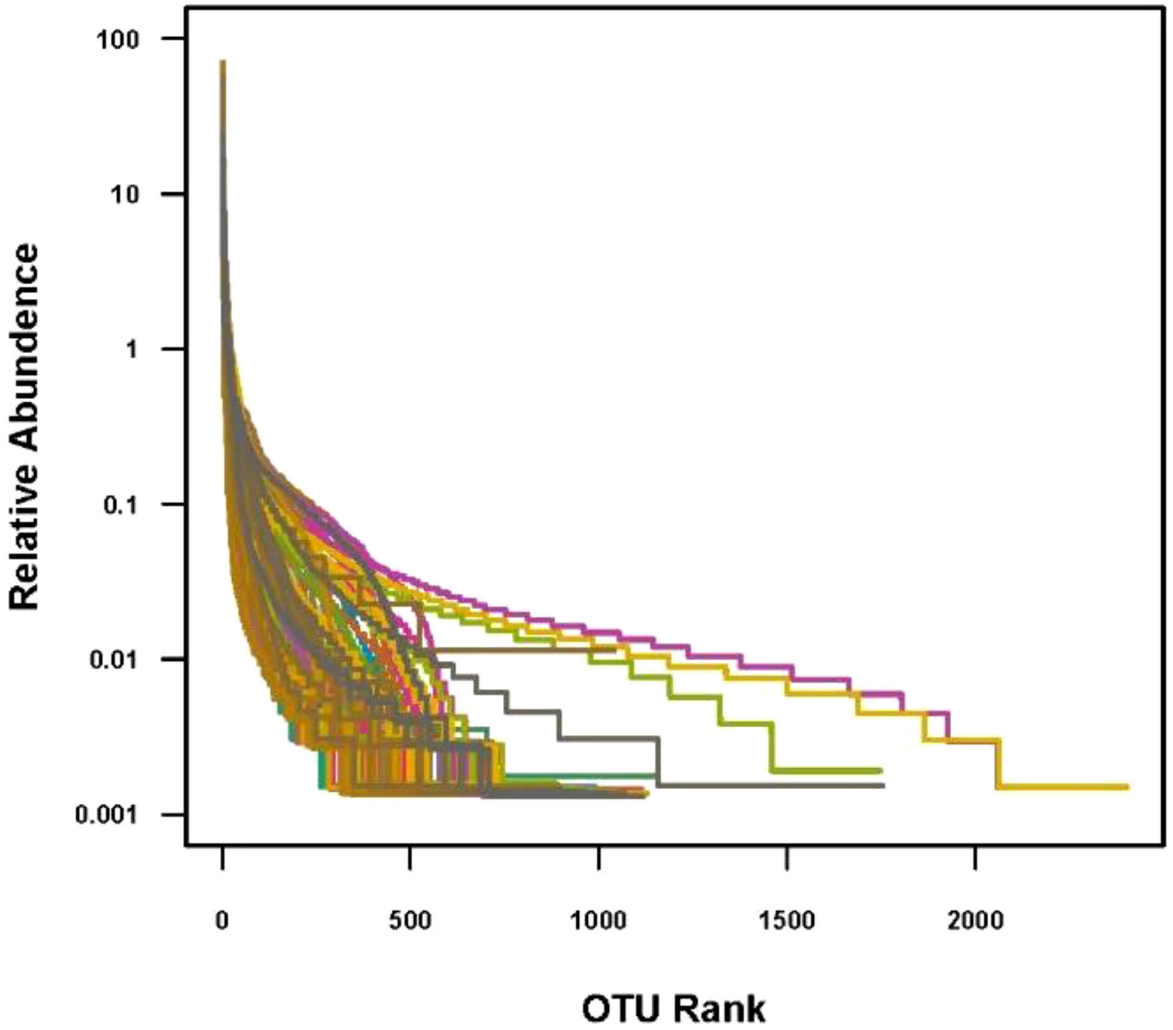
OTU rank. In the abscissa, OTUs are sorted according to the number of sequences contained from the most to the least. The ordinate represents the relative abundance of the OTU.

The data volume of clean tags for samples is between 12,266 and 78,875 bp. The data volume of clean tags obtained by removing chimeras is between 8,817 and 76,313 bp. The average length of valid tags is between 406.47 and 425.23 bp. The OTUs analysis showed a long tail in the rank abundance curves, indicating that the majority of OTUs were at low abundance, and all the OTUs were evenly distributed (**Figure 2**).

### 3.2 Comparison of preoperative group with the postoperative group

#### 3.2.1 Alpha diversity

**Figure 3:**
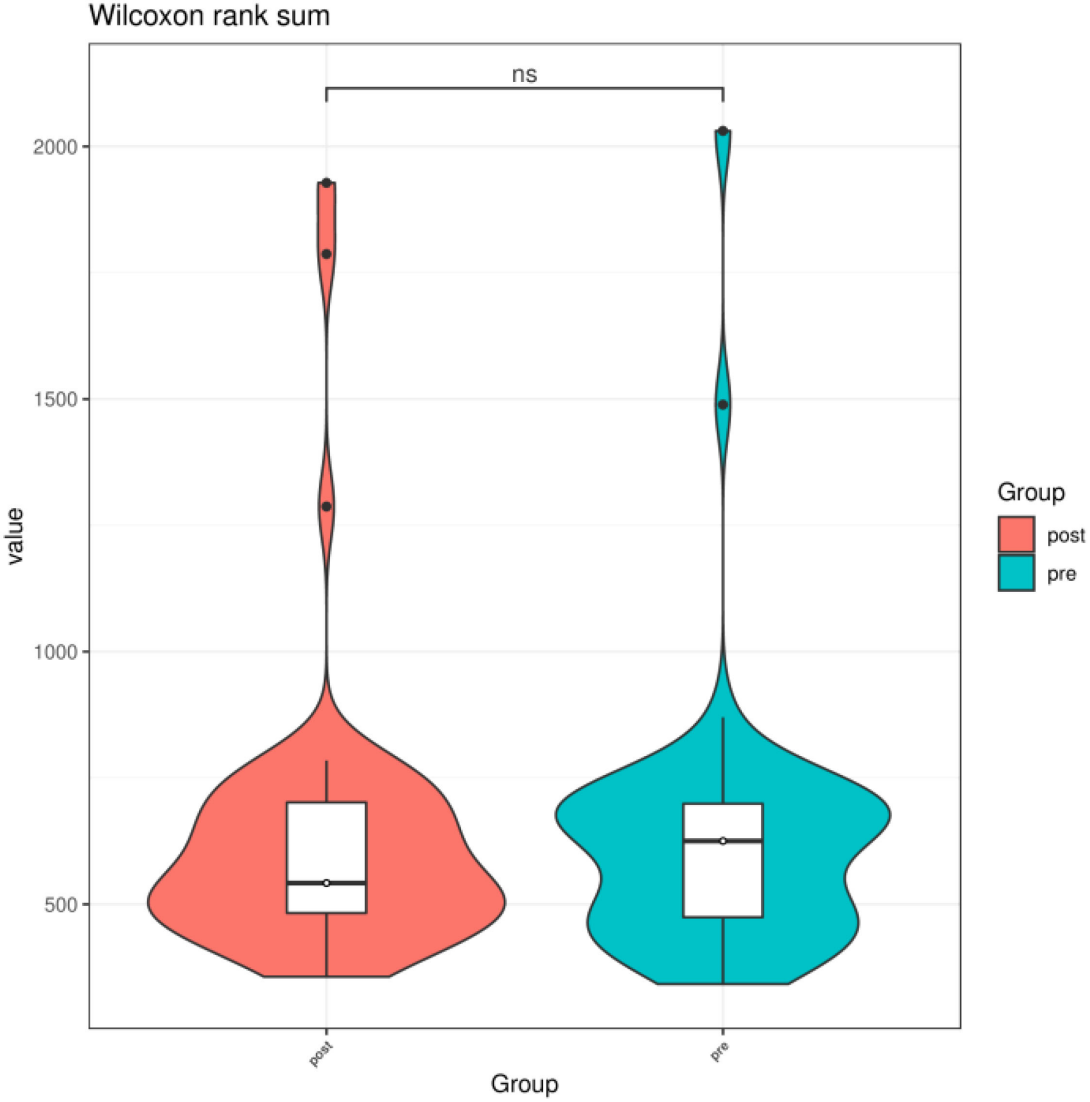
Chao1 index of gut microbiota between the preoperative group and the postoperative group. ns indicates *P* > 0.05; pre indicates the preoperative group; post indicates the postoperative group.

**Figure 4:**
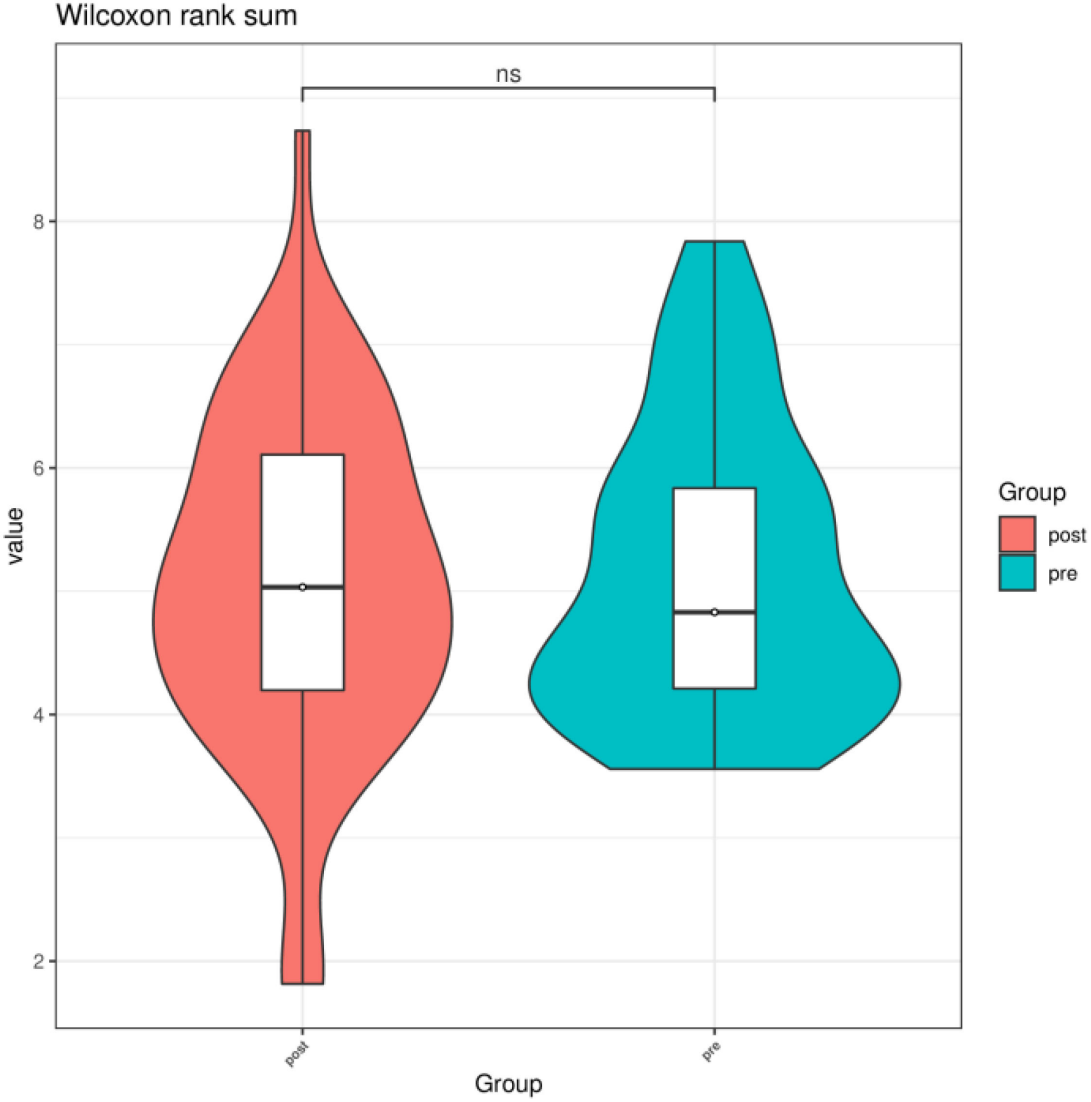
Shannon index of gut microbiota between the preoperative group and the postoperative group. ns indicates *P* > 0.05; pre indicates the preoperative group; post indicates the postoperative group.

**Figure 5:**
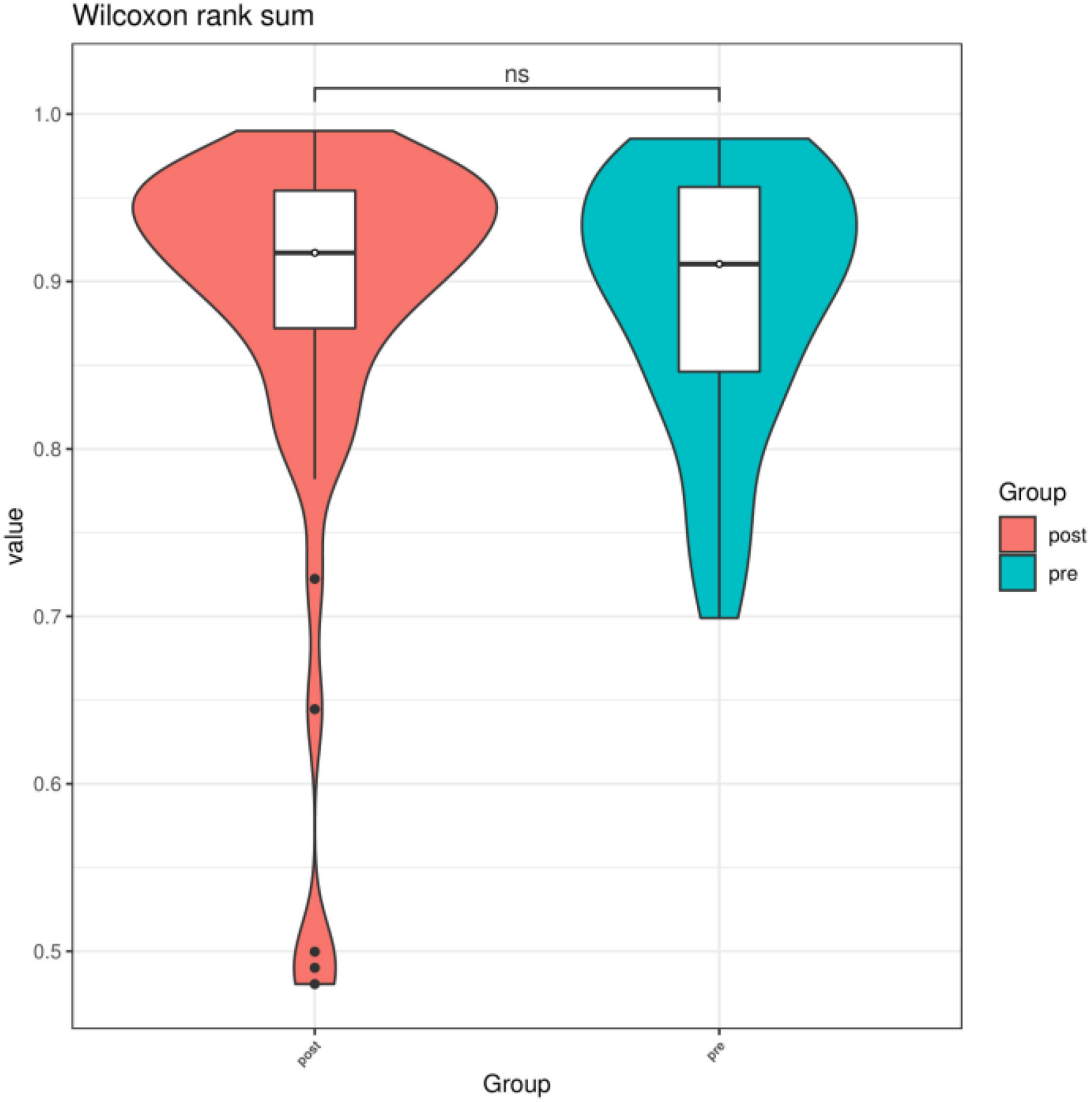
Simpson index of gut microbiota between the preoperative group and the postoperative group. ns indicates *P* > 0.05; pre indicates preoperative group; post indicates the postoperative group.

We compared the Chao1 index, Shannon index, and Simpson index between the preoperative group and the postoperative group. There was no statistical difference in the Chao1 index, Shannon index, and Simpson index between the preoperative group and the postoperative group (*P* > 0.05) (**Figures 3**–**5**).

#### 3.2.2 Beta diversity

**Figure 6:**
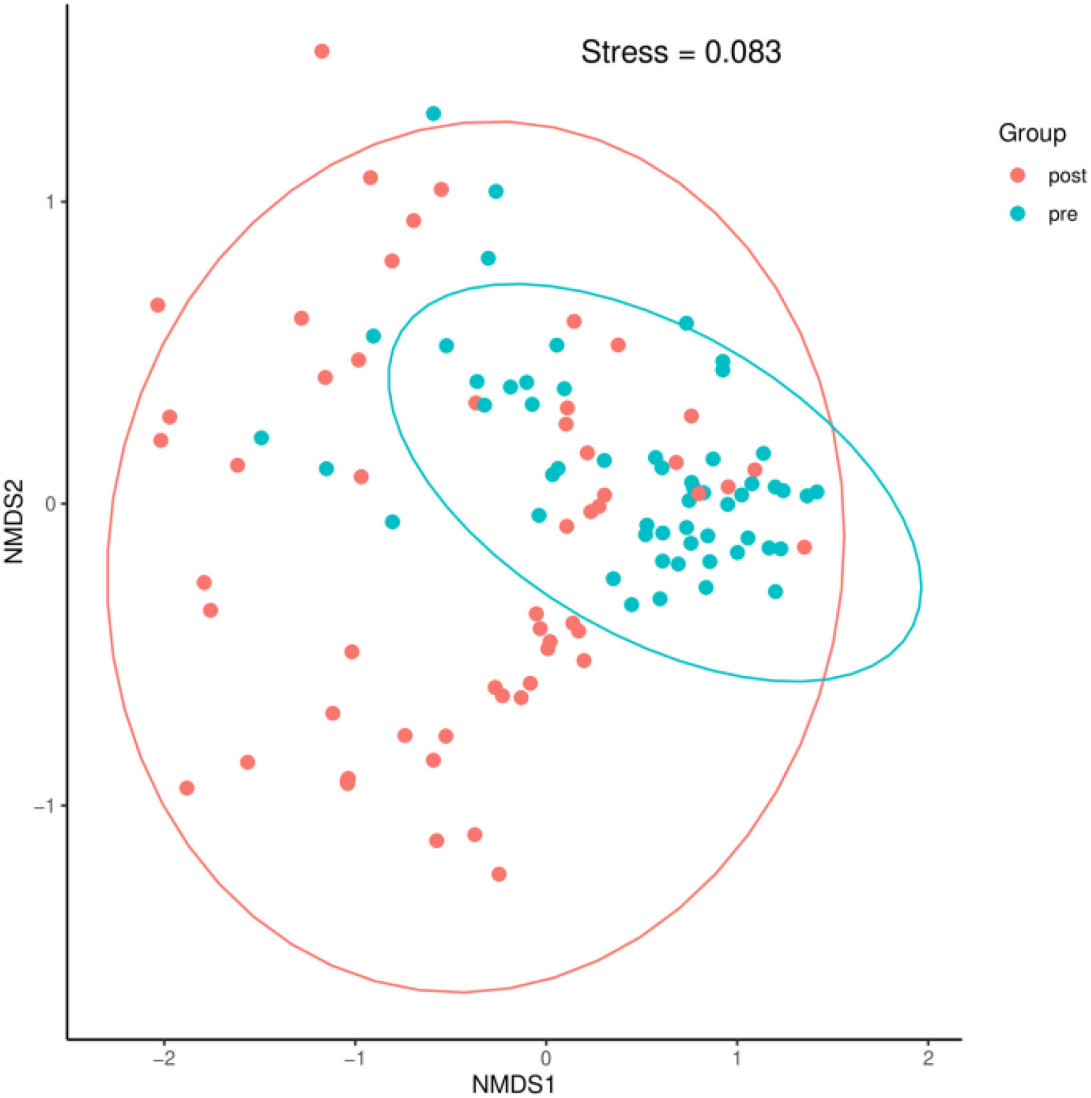
A Bray–Curtis NMDS plot. pre indicates the preoperative group; post indicates the postoperative group.

A Bray–Curtis NMDS plot (**Figure 6**) could distinguish samples of preoperative group from the postoperative group significantly. ANOSIM statistical analysis based on Bray–Curtis distance algorithm showed that the R^2^ value of grouping factors was 0.127, *P* = 0.001.

#### 3.2.3 Intestinal microflora structure

**Figure 7:**
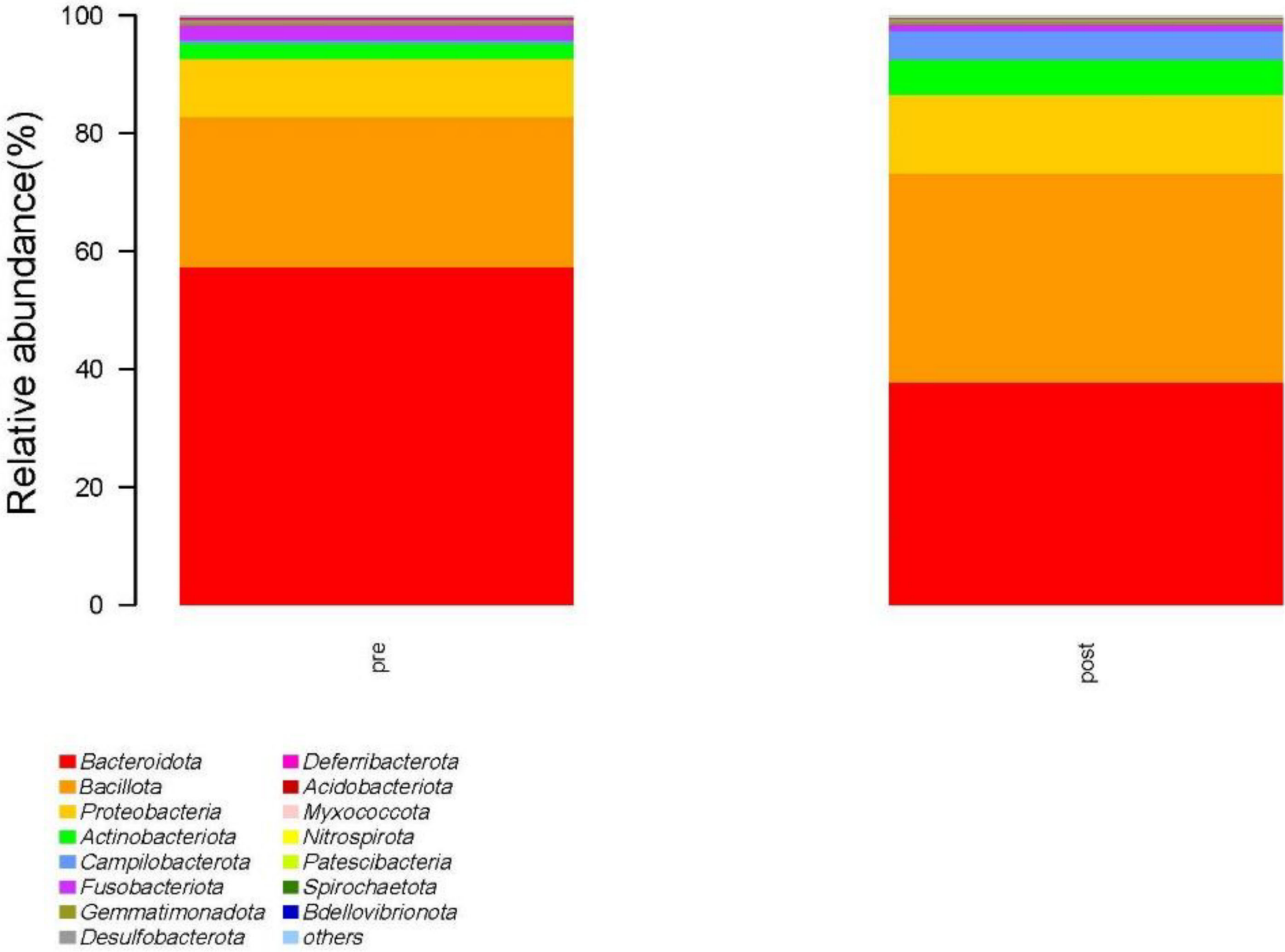
Species with relative abundance of top 15 at the phylum level. pre indicates the preoperative group; post indicates the postoperative group.

**Figure 8:**
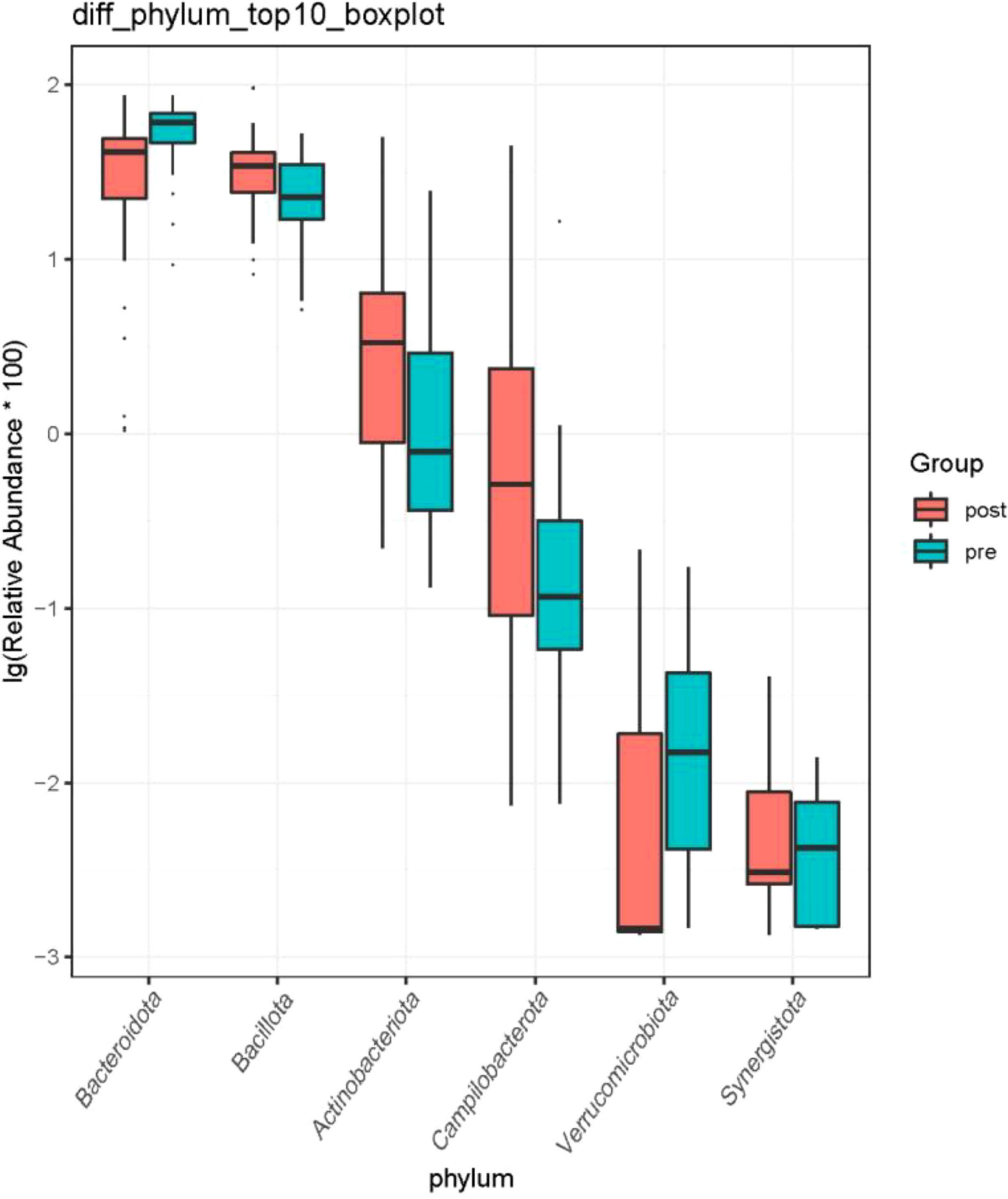
Difference in relative abundance of top 10 bacteria at the phylum level in the preoperative group and the postoperative group. pre indicates the preoperative group; post indicates the postoperative group.

At the phylum level, the species with relative abundance of top 15 are shown in **Figure 7**. The intestinal microbiota was dominated by *Bacteroidota*, *Bacillota*, *Proteobacteria*, *Actinobacteria*, and *Campilobacterota*. Compared with the preoperative group, the postoperative group had mainly decreased abundance of *Bacteroidota*, *Synergistota*, and *Verrucomimicrobiota* (**Figure 8**, all *P* < 0.05). Compared with the preoperative group, the postoperative group had mainly increased abundance of *Campylobacter*, *Actinobaciota*, and *Bacillota* (**Figure 8** all, *P* < 0.05).

**Figure 9:**
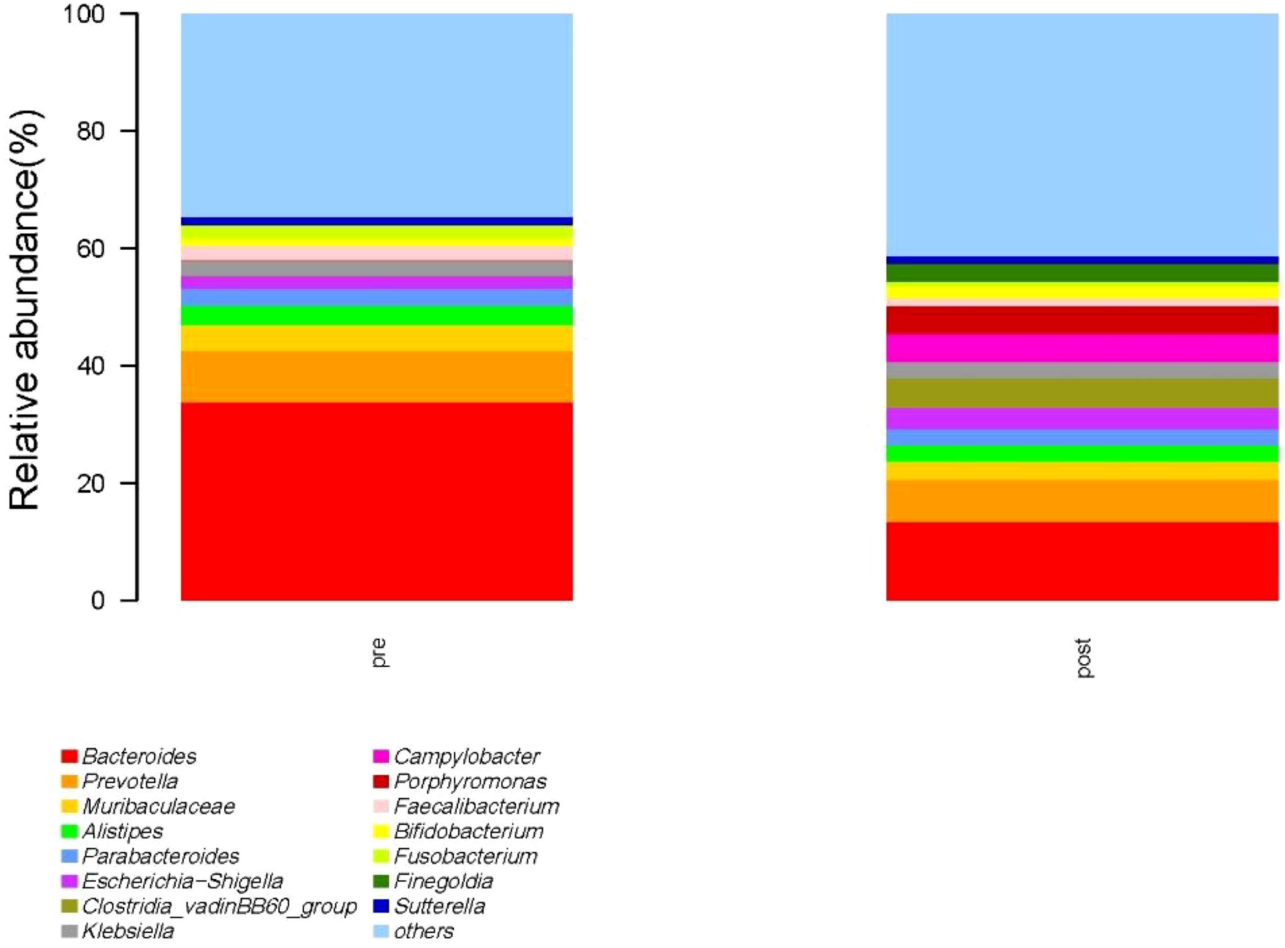
Species with relative abundance of top 15 at the genus level. pre indicates the preoperative group; post indicates the postoperative group.

**Figure 10:**
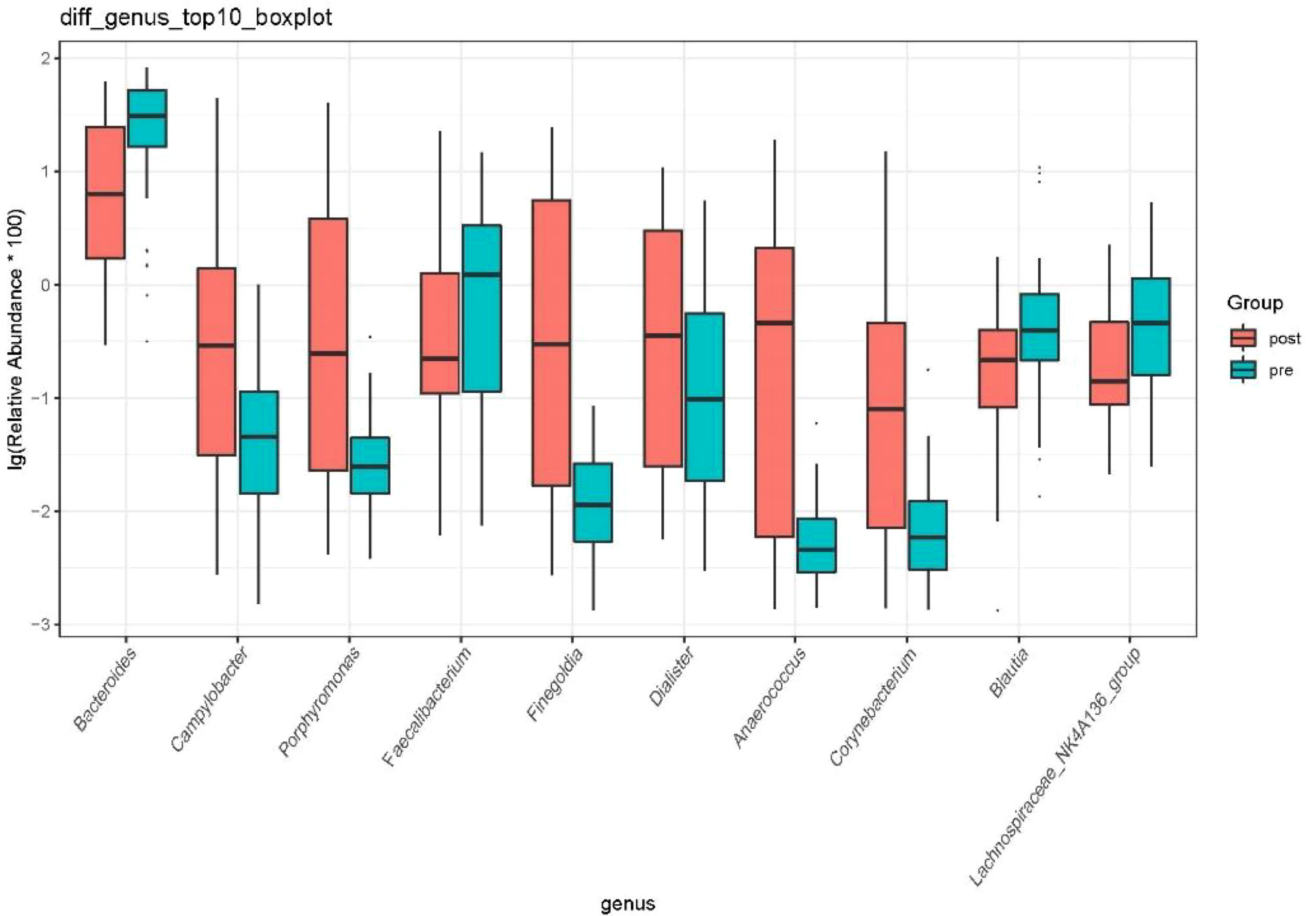
Difference in relative abundance of top 10 bacteria at the genus level in the preoperative group and the postoperative group. pre indicates the preoperative group; post indicates the postoperative group.

At the genus level, the species with relative abundance of top 15 are shown in **Figure 9**. The intestinal microbiota was dominated by *Bacteroides*, *Prevotella*, *Muribaculareae*, *Alistipes*, and *Parabacteroides*. Compared with the preoperative group, the postoperative group had mainly decreased abundance of *Bacteroides*, *Faecalibacterium*, *Blautia*, and *Lachnospiraceae_NK4A136_*group (**Figure 10**, all *P* < 0.05). Compared with the preoperative group, the postoperative group had mainly increased abundance of *Campylobacter*, *Porphyromona*, *Finegoldia*, *Dialister*, *Anaerococcus*, and *Corynebacterium* (**Figure 10**, all *P* < 0.05).

## 4 Discussion

There are approximately 10^13^~10^14^ microorganisms in the human gut, with a total genome size approximately 100 times that of the human genome (22). Literature research shows that the changes of intestinal microbial community composition are closely related to diabetes, inflammatory bowel disease, obesity, and other diseases (23, 24). Various microorganisms living in the human body actively participate in physiological processes such as immunity, digestion, and toxin degradation in the body and have significant effects on human development and health (9). However, because of the characteristics of strong metabolism, strong diffusion ability, small individual size, and high mutational rates, it is difficult for traditional pure culture methods to obtain an accurate and comprehensive understanding of the overall situation of microorganisms (12).

Microbial 16SrDNA high-throughput sequencing explores the relationship between microbes and organisms and between microbes and the natural environment from top to bottom by looking at the genetic information of all microbes in the environment as a whole. It breaks through the technical bottleneck of difficult cultivation of microorganisms and can also be combined with the use of bioinformatics methods to reveal the laws of interaction between microorganisms and the environment; the same is true among microorganisms, greatly broadening the research methods and ideas of microbiology, opening up a new way to re understand the functions and ecological characteristics of microorganisms from the perspective of microbial community structure.

The reconstruction of the digestive tract after surgery for GC has fundamentally changed the intestinal microenvironment, including intestinal oxygen availability, pH, and food transport time. However, the results of this study showed that there was no statistical difference in the Chao1 index, Shannon index, and Simpson index between the preoperative group and the postoperative group (*P* > 0.05). This indicates that surgery does not affect the diversity of fecal microbial species in patients with GC. This result is inconsistent with the results of a study in Japan. According to Erawijantari’s research in Japan, α diversity of the postoperative GC group was significantly higher than that of the normal group (23). The possible reason why this result is inconsistent with this study is that the selection of the control group is different. We selected preoperative patients with GC as the control group, whereas the Japanese study selected normal people as the control group. GC itself has had an impact on the diversity of intestinal flora.

Currently, there are few studies on the impact of GC surgery on the intestinal flora of patients. 16S rRNA gene sequencing was used to evaluate differences in gut microbiota among patients with GC before and after the gastrectomy by comparing gut microbiota β diversity and structure composition at different taxonomic levels. Compared with the preoperative group, the postoperative group had mainly increased abundance of *Campylobacter*, *Actinobaciota*, and *Bacillota* (**Figure 8**, all *P* < 0.05). Compared with the preoperative group, the postoperative group had mainly decreased abundance of *Bacteroides*, *Faecalibacterium*, *Blautia*, and *Lachnospiraceae_NK4A136_*group (**Figure 10**, all *P* < 0.05). Compared with the preoperative group, the postoperative group had mainly increased abundance of *Campylobacter*, *Porphyromona*, *Finegoldia*, *Dialister*, *Anaerococcus*, and *Corynebacterium* (**Figure 10**, all *P* < 0.05).

*Bacteroides*, *Lachnospiraceae_NK4A136_*group, and *Faecalibacteriu* are potentially beneficial bacteria. Studies have shown that Bacteroides participate in a variety of important metabolic activities in the intestinal tract, including carbohydrate fermentation, the use of nitrogen-containing substances, and the biological transformation of bile acids and other steroids. In addition, *Bacteroides* helps the host resist colonization of intestinal pathogens and volatilize immune regulation, preventing invasion by invasive pathogens (24, 25). *Lachnospiraceae_NK4A136_*group and *Clostridium pratense* in *Faecalibacteriu* are one of the most important bacteria in the human intestinal flora. The acetic acid and butyric acid produced by fermentation have anti-inflammatory effects and maintain bacterial enzyme activity (25, 26). The decrease in abundance of *Lachnospiraceae_NK4A136_*group and *Faecalibacteriu* may be due to surgical stress, leading to GI physiological changes, such as inhibition of gastric acid release, changes in GI motility, and increased production of bicarbonate in the duodenum. These changes are not conducive to the survival, adhesion, and replication of *Bacteroides* and *Spirillum*, resulting in a decrease in their number.

*Campylobacter* is a typical aerobic bacterium. *Corynebacterium* and *Dialister* are aerobic or facultative anaerobic bacteria. *Porphyromona* is a common microorganism associated with oral diseases. The reconstruction of the digestive tract after surgery for GC has changed the intestinal environment, possibly providing conditions for the growth of aerobic or facultative anaerobes, as well as creating opportunities for the translocation of oral microorganisms (27, 28). Many research studies have reported that *Finegoldia* and *Anaerococus* are highly pathogenic conditional pathogens, most of which belong to the Gram-negative group. In addition, *Finegoldia* is one of the most common pathogens in the etiology of post-prosthetic implantation-associated septic arthritis (29, 30), which is enriched after GC surgery, whereas the reduction of *Bacteroides* and *Laclobacteria* increases the chances of infection in patients.

Unfortunately, our study did not detect and analyze changes in the oral flora of patients with GC during the perioperative period. In a systematic review by Maksimaityte et al. (31), it was mentioned that gastrectomy-induced dysbiosis is characterized by an increase in the number of typical oral bacteria, an increase in the number of oxygen-resistant bacteria (aerobic/parthenogenetic anaerobic), and an increase in the number of bile acid–converting bacteria. Due to a certain association between our oral and intestinal flora, the relative abundance of oral colonization in the fecal matter increases when the intestinal flora decreases and is associated with patient prognosis (32). Therefore, certain oral colonizing bacteria might be able to enter the intestinal lumen by aiding the food digestion process or the gastric tube route, which could be a potential new idea for flora regulation.

This study has some limitations. First, this is a single-center study, and the results may not be generalizable to the entire country. Second, the sample size in this study was small and limited in scope, which warrants further research in a larger sample.

## Data Availability

The original contributions presented in the study are included in the article/supplementary material. Further inquiries can be directed to the corresponding author.
